# A new species of terrestrial-breeding frog (Amphibia, Craugastoridae, *Pristimantis*) from high elevations of the Pui Pui Protected Forest in central Peru

**DOI:** 10.3897/zookeys.660.11394

**Published:** 2017-03-07

**Authors:** Edgar Lehr, Rudolf von May

**Affiliations:** 1 Department of Biology, Illinois Wesleyan University, 303 E Emerson, Bloomington, IL 61701, USA; 2 Department of Ecology and Evolutionary Biology, Museum of Zoology, University of Michigan, 2051 Ruthven Museums Building, 1109 Geddes Ave., Ann Arbor, MI 48109, USA

**Keywords:** Andes, DNA barcoding, frogs, molecular phylogeny, montane forest, *Pristimantis attenboroughi* new species, Puna

## Abstract

We describe a new species of *Pristimantis* from upper montane forests and high Andean grasslands of the Pui Pui Protected Forest and its close surroundings, Región Junín, central Peru. The description of the new species is based on 34 specimens found at elevations between 3400 and 3936 m a.s.l. *Pristimantis attenboroughi* **sp. n.** is characterized by a snout–vent length of 14.6–19.2 mm in adult males (n = 21), 19.2–23.0 mm in adult females (n = 10), and is compared morphologically and genetically with other taxonomically and biogeographically relevant species of *Pristimantis*. The new species is characterized by having narrow digits that lack circumferential grooves, irregularly shaped, discontinuous dorsolateral folds, and absence of both tympanic membrane and tympanic annulus. The high similarity in morphology between *P. attenboroughi* **sp. n.** and members of the Andean genera *Phrynopus* and *Bryophryne* provides an example for convergent evolution, and highlights the importance of using molecular data to justify generic assignment. *Pristimantis attenboroughi* **sp. n.** is most similar to *Phrynopus chaparroi* from the Región Junín, suggesting that the generic placement of this species needs to be revised. Phylogenetically the new species belongs to the *Pristimantis danae* species Group, a clade that includes several *Pristimantis* species distributed in the montane forests of central Peru, including *P. albertus*, *P. aniptopalmatus*, *P. ornatus*, and *P. stictogaster*.

## Introduction

**Figure 1.**
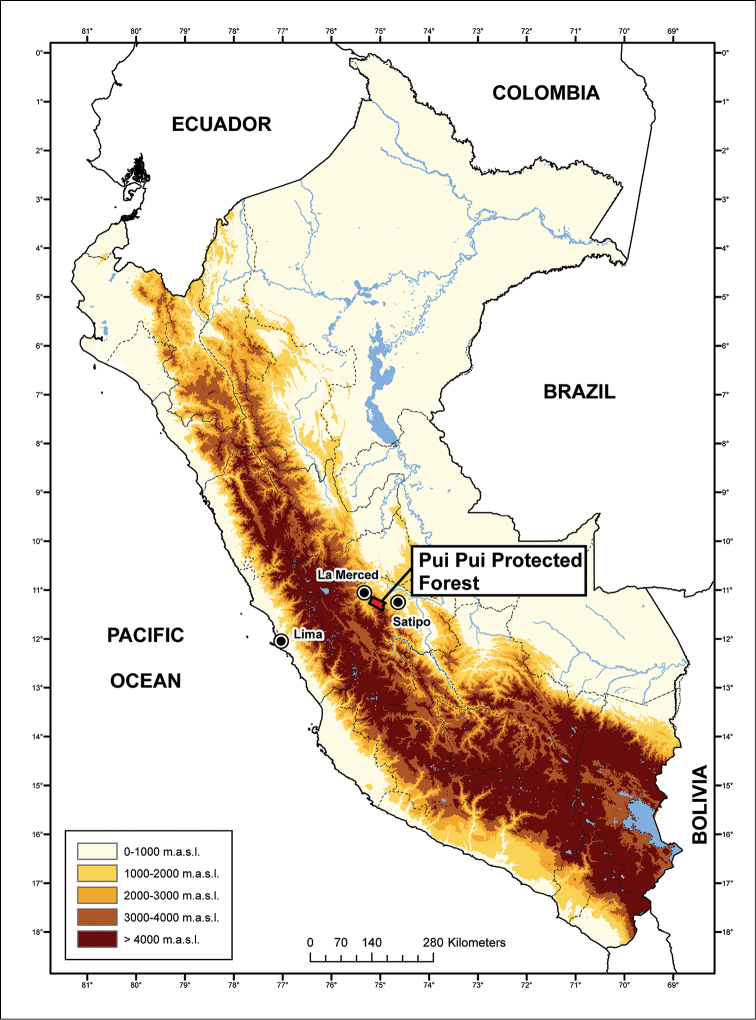
Map of Peru with the Pui Pui Protected Forest indicated in red.

The Pui Pui Protected Forest (Bosque de Protección Pui Pui, hereafter PPPF, Fig. 1) is located in the Selva Central of Peru and is one of twelve natural protected areas with different levels of legal protection such as national parks, national sanctuaries, and national reserves in the regions of Pasco and Junín (Sernanp 2010). The PPPF, located in the Región Junín, was established in 1985 and covers 60,000 hectares encompassing montane forest (30%) and high Andean grassland (Puna; 70%) habitats (Sernanp 2010). The area protects the upper watershed of several rivers and includes elevations between 1700 and 4500 m a.s.l. (Sernanp 2010).

In 2012–2014, we conducted herpetological surveys in montane forests and Puna of the PPPF to catalog the amphibian and reptile species and to evaluate their conservation status. As a result, we found several new species of frogs (Craugastoridae) as well as new species of lizards (Gymnophthalmidae). All new species were compared morphologically and genetically with other taxonomically and biogeographically relevant taxa mostly from Ecuador, Peru, and Bolivia. Herein we describe a new species of *Pristimantis* from upper montane and Puna habitats collected between 2012 and 2013.

## Materials and methods

**Fieldwork.** Because of its remote location, the PPPF is difficult to reach and is only accessible through a few entrances located ca. 1–2 days of walking distance from the nearest villages. The upper montane forests and Puna of the PPPF were reached from Toldopampa (11°30'15.4"S, 74°55'32.7"W, 3670 m a.s.l., ca. 45 km SW from Satipo) with the help of local guides by walking in 1.5 days (ca. 11 km airline). In 2012 fieldwork was conducted between May 8 and 21 by EL and RvM, and in 2013 between June 21 and July 8 by EL, J. Moravec, and J.C. Cusi. Amphibians were preserved in 96% ethanol and stored in 70% ethanol. Deposited eggs were stored in 70% ethanol.

**Morphological characters.** The format for the description follows Lynch and Duellman (1997), except that the term dentigerous processes of vomers is used instead of vomerine odontophores (Duellman et al. 2006), and diagnostic characters are those of Duellman and Lehr (2009). Taxonomic classification follows Hedges et al. (2008), except that we followed Pyron and Wiens (2011) for family placement and Padial et al. (2014) for names of *Pristimantis* species groups. Sex and maturity of specimens were identified by observing gonads through dissections. Specimens were considered juveniles when gonads were too small to distinguish between sexes. The tympanic region of two specimens (MUSM 31199, NMP6V 75534) was opened to see if a tympanic annulus is present under the skin. We measured the following variables to the nearest 0.1 mm with digital calipers under a stereomicroscope: snout–vent length (SVL, straight length distance from tip of snout to vent), tibia length (TL, distance from the knee to the distal end of the tibia), foot length (FL, distance from proximal margin of inner metatarsal tubercle to tip of Toe IV), head length (HL, from angle of jaw to tip of snout), head width (HW, at level of angle of jaw), horizontal eye diameter (ED), interorbital distance (IOD), upper eyelid width (EW), internarial distance (IND), eye–nostril distance (E-N, straight line distance between anterior corner of orbit and posterior margin of narial opening), and egg diameter. Fingers and toes are numbered preaxially to postaxially from I–IV and I–V, respectively. We compared the lengths of toes III and V by adpressing both toes against Toe IV; lengths of fingers I and II were compared by adpressing the fingers against each other. All drawings were made by EL using a stereomicroscope and a camera lucida. Photographs taken by EL and RvM were used for descriptions of coloration in life. Comparisons of congeners focus on species in similar habitats from Ecuador and Peru and those with close phylogenetic relationships as recovered in our phylogenetic trees. Information on species for comparative diagnoses was obtained from Duellman and Lehr (2009) and from original species descriptions. For specimens examined see Appendix. Codes of collections are: MUSM = Universidad Nacional Mayor de San Marcos, Lima, Peru; NMP6V = National Museum Prague, Prague, Czech Republic; UMMZ = University of Michigan Museum of Zoology, Ann Arbor, USA. Field number code is: IWU = Illinois Wesleyan University, Bloomington, USA. Conservation status was evaluated using the criteria in IUCN (2001). Maps were designed with ArcGIS 10.0 by J.C. Cusi.

**Molecular phylogenetic analysis.** The phylogenetic position of the new species with respect to other morphologically similar species was determined through analysis of DNA sequence data. This analysis included two mitochondrial genes, 16S rRNA (16S) and 12S rRNA (12S). We used tissue samples from specimens collected in central Peru (Región Junín) to obtain DNA sequences for the new species and several other *Pristimantis* species (Table 1). Additionally, we downloaded selected sequences of morphologically similar taxa (*Bryophyrne*, *Lynchius*, *Phrynopus*, *Oreobates*) distributed at high elevations (> 2000 m a.s.l.) from Genbank (Table 1). We included *Hamptophryne boliviana*, *Ischnocnema guentheri*, and *Bufo melanostictus* as outgroup taxa (Padial et al. 2014).

**Table 1.** GenBank accession numbers for taxa and genes sampled in this study.

Taxon	16S	12S	Voucher_Nbr	Reference
*Bryophryne bakersfield*	KT276289	na	MHNC5999	Chaparro et al. 2015
*Bryophryne bakersfield*	KT276287	KT276281	MHNC6022	Chaparro et al. 2015
*Bryophryne bakersfield*	KT276290	KT276282	MHNC6023	Chaparro et al. 2015
*Bryophryne bakersfield*	KT276291	KT276283	MHNC6007	Chaparro et al. 2015
*Bryophryne bakersfield*	KT276288	KT276284	MHNC6009	Chaparro et al. 2015
*Bryophryne bustamantei*	KT276293	KT276286	MHNC6019	Chaparro et al. 2015
*Bryophryne cophites*	EF493537	EF493537	KU173497	Heinicke et al. 2007
*Bufo melanostictus*	FJ882791	FJ882791	VUB 0052	Van Bocxlaer et al. 2009
*Hamptophryne boliviana*	DQ283438	DQ283438	na	Frost et al. 2006
*Ischnocnema guentheri*	EF493533	EF493533	na	Heinicke et al. 2007
*Lynchius flavomaculatus*	EU186667	EU186667	KU218210	Hedges et al. 2008
*Lynchius nebulanastes*	EU186704	EU186704	KU181408	Hedges et al. 2008
*Lynchius oblitus*	AM039640	AM039708	MUSM19914	Lehr et al. 2005, Motta et al. 2016
*Lynchius oblitus*	AM039639	AM039707	MTD45954	Lehr et al. 2005, Motta et al. 2016
*Lynchius parkeri*	EU186705	EU186705	KU181307	Hedges et al. 2008
*Lynchius simmonsi*	JF810004	JF809940	QZ41639	Padial et al. 2014
*Oreobates amarakaeri*	JF809996	JF809934	MHNC6975	Padial et al. 2014
*Oreobates ayacucho*	JF809970	JF809933	MNCN_IDlR5024	Padial et al. 2014
*Oreobates cruralis*	EU186666	EU186666	KU215462	Hedges et al. 2008
*Oreobates gemcare*	JF809960	JF809930	MHNC6687	Padial et al. 2014
*Oreobates granulosus*	EU368897	JF809929	MHNC3396	Padial et al. 2014
*Phrynopus auriculatus*	EF493708	EF493708	KU291634	Heinicke et al. 2007
*Phrynopus barthlenae*	AM039653	AM039721	SMF81720	Lehr et al. 2005
*Phrynopus bracki*	EF493709	EF493709	USNM286919	Heinicke et al. 2007
*Phrynopus bufoides*	AM039645	AM039713	MUSM19860	Lehr et al. 2005
*Phrynopus heimorum*	AM039635	AM039703	MTD45621	Lehr et al. 2005
*Phrynopus heimorum*	AM039636	AM039704	MTD45622	Lehr et al. 2005
*Phrynopus horstpauli*	AM039651	AM039719	MTD44333	Lehr et al. 2005
*Phrynopus horstpauli*	AM039647	AM039715	MTD44334	Lehr et al. 2005
*Phrynopus kauneorum*	AM039650	AM039718	MTD44332	Lehr et al. 2005
*Phrynopus kauneorum*	AM039655	AM039723	MUSM20595	Lehr et al. 2005
*Phrynopus pesantesi*	AM039656	AM039724	MTD45072	Lehr et al. 2005
*Phrynopus tautzorum*	AM039652	AM039720	MUSM20613	Lehr et al. 2005
*Phrynopus tribulosus*	EU186725	EU186707	KU291630	Hedges et al. 2008
*Pristimantis acuminatus*	EU130579	na	QCAZ19664	Elmer et al. 2007
*Pristimantis albertus*	EU186695	EU186695	KU291675	Hedges et al. 2008
*Pristimantis albertus*	KY594749	na	RVM41_14	This study
*Pristimantis albertus*	KY594750	na	RVM42_14	This study
*Pristimantis albertus*	KY594751	na	RVM527	This study
*Pristimantis altamazonicus*	EF493670	EF493670	KU215460	Heinicke et al. 2007
*Pristimantis altamazonicus*	DQ195449	na	MC11717	Mahecha et al., unpublished
*Pristimantis aniptopalmatus*	EF493390	EF493390	KU291627	Heinicke et al. 2007
*Pristimantis aniptopalmatus*	EU186694	EU186694	KU291666	Padial et al. 2014
*Pristimantis attenboroughi* sp. n.	KY594752	na	MUSM31186	This study
*Pristimantis attenboroughi* sp. n.	KY594753	KY594761	NMP6V75522	This study
*Pristimantis attenboroughi* sp. n.	KY594754	KY594762	NMP6V75524	This study
*Pristimantis attenboroughi* sp. n.	KY594755	KY594763	NMP6V75525	This study
*Pristimantis attenboroughi* sp. n.	KY594756	KY594764	NMP6V75528	This study
*Pristimantis attenboroughi* sp. n.	KY594757	na	NMP6V75529	This study
*Pristimantis aureoventris*	JQ742152	na	VUB3748	Kok et al. 2012
*Pristimantis bipunctatus*	EF493702	EF493702	KU291638	Heinicke et al. 2007
*Pristimantis bipunctatus*	KY594758	na	MUSM31179	This study
Pristimantis cf. mendax	KY628996	na	MUSM31157	This study
Pristimantis cf. mendax	EU186659	na	MTD45080	Hedges et al. 2008
*Pristimantis croceoinguinis*	KY594759	na	MUSM31154	This study
*Pristimantis cruciocularis*	EU186656	EU186656	KU291673	Hedges et al. 2008
*Pristimantis cruciocularis*	KY594760	na	NMP6V75535	This study
*Pristimantis danae*	EU192270	na	MNCN44234	Padial and De la Riva 2009
*Pristimantis diadematus*	EU186668	EU186668	KU221999	Hedges et al. 2008
*Pristimantis llojsintuta*	EU712641	na	MNCNDNA7314	Padial et al. 2009
*Pristimantis melanogaster*	EF493664	EF493826	na	Heinicke et al. 2007
*Pristimantis orestes*	EF493388	EF493388	KU218257	Heinicke et al. 2007
*Pristimantis ornatus*	EU186660	EU186660	MTD45073	Hedges et al. 2008
*Pristimantis petrobardus*	EF493367	EF493825	KU212293	Heinicke et al. 2007
*Pristimantis platydactylus*	EU712653	na	MNCNDNA3943	Padial et al. 2009
*Pristimantis platydactylus*	EU712671	na	MNCNDNA4138	Padial et al. 2009
*Pristimantis platydactylus*	EU712718	na	MNCNDNA6377	Padial et al. 2009
*Pristimantis pluvialis*	KX155577	na	CORBIDI_11862	Shepack et al. 2016
*Pristimantis pluvialis*	KX155578	na	CORBIDI_16695	Shepack et al. 2016
*Pristimantis reichlei*	EF493707	EF493707	MUSM9267	Padial et al. 2014
*Pristimantis rhabdocnemus*	EU186706	EU186724	KU291651	Hedges et al. 2008
*Pristimantis rhabdolaemus*	EF493706	EF493706	KU173492	Heinicke et al. 2007
*Pristimantis sagittulus*	EF493705	EF493705	KU291635	Duellman and Hedges 2005
*Pristimantis schultei*	EF493681	na	KU212220	Heinicke et al. 2007
*Pristimantis simonbolivari*	EF493671	EF493671	KU218254	Heinicke et al. 2007
*Pristimantis simonsii*	EU186665	EU186665	KU212350	Hedges et al. 2008
*Pristimantis skydmainos*	EF493393	EF493393	MUSM10071	Heinicke et al. 2007
*Pristimantis* sp.	AM039658	na	MTD45201	Lehr et al. 2005
*Pristimantis stictogaster*	EF493704	EF493704	KU291659	Heinicke et al. 2007
*Pristimantis toftae*	EF493353	EF493353	KU215493	Heinicke et al. 2007
*Pristimantis toftae*	EU192294	na	MNCN43246	Padial and De la Riva 2009
*Pristimantis wiensi*	EF493668	EF493377	KU219796	Heinicke et al. 2007

Extraction, amplification, and sequencing of DNA followed protocols previously used for Neotropical terrestrial breeding frogs (Lehr et al. 2005, Hedges et al. 2008). We used the 16SA (forward) primer (5’-3’ sequence: CGCCTGTTTATCAAAAACAT) and the 16SB (reverse) primer (5’-3’ sequence: CCGGTCTGAACTCAGATCACGT) to amplify a fragment of the 16S gene (Palumbi et al. 1991), and we employed the following thermocycling conditions to amplify DNA using the polymerase chain reaction (PCR): 1 cycle of 96°C/3 min; 35 cycles of 95°C/30 s, 55°C/45 s, 72°C/1.5 min; 1 cycle 72°C/7 min. Additionally, we used the L25195 (forward) primer (5’-3’ sequence: AAACTGGGATTAGATACCCCACTA) and the H2916 (reverse) primer (5’-3’ sequence: GAGGGTGACGGGCGGTGTGT) to amplify a fragment of the 12S gene (Palumbi et al. 1991, Vences et al. 2000), and we employed the following thermocycling conditions to amplify DNA using PCR: 1 cycle of 94°C/1.5 min; 35 cycles of 94°C/45 s, 50°C/1 min., 74°C/2 min; 1 cycle 72°C/10 min. We completed the cycle sequencing reactions by using the corresponding PCR primers and the BigDye Terminator 3.1 (Applied Biosystems), and obtained sequence data by running the purified reaction products in an ABI 3730 Sequence Analyzer (Applied Biosystems). The newly obtained sequences are deposited in GenBank (Table 1).

Geneious R6, version 6.1.8 (Biomatters 2013; http://www.geneious.com/) was used to align the sequences. Within Geneious, we used the MAFFT, version 7.017 (Katoh and Standley 2013) alignment program. Prior to conducting phylogenetic analysis, we used PartitionFinder, version 1.1.1 (Lanfear et al. 2012) to select the appropriate models of nucleotide evolution and used the Bayesian information criterion (BIC) to determine the best partitioning scheme and substitution model for each gene. According to PartitionFinder, the best scheme included one partition combining both 12S and 16S and the best model of nucleotide substitution was GTR + I + Γ. Phylogenetic analysis was done using Maximum Likelihood (ML) approach using RaxML version 8.2.4 (Stamatakis 2006), where the “f-a” function was employed to conduct a bootstrap analysis and search for the optimal likelihood tree. Our analysis included 82 terminals and a 922 bp concatenated alignment that included the 16S and 12S dataset. The GTR + I + Γ model of nucleotide substitution was used to perform 200 trees searches; node support was assessed using 1000 bootstrap replicates. Additionally, we used the R package ‘APE’ (Paradis et al. 2004) to estimate uncorrected p-distances (i.e., the proportion of nucleotide sites at which any two sequences are different).

## Results

**Figure 2.**
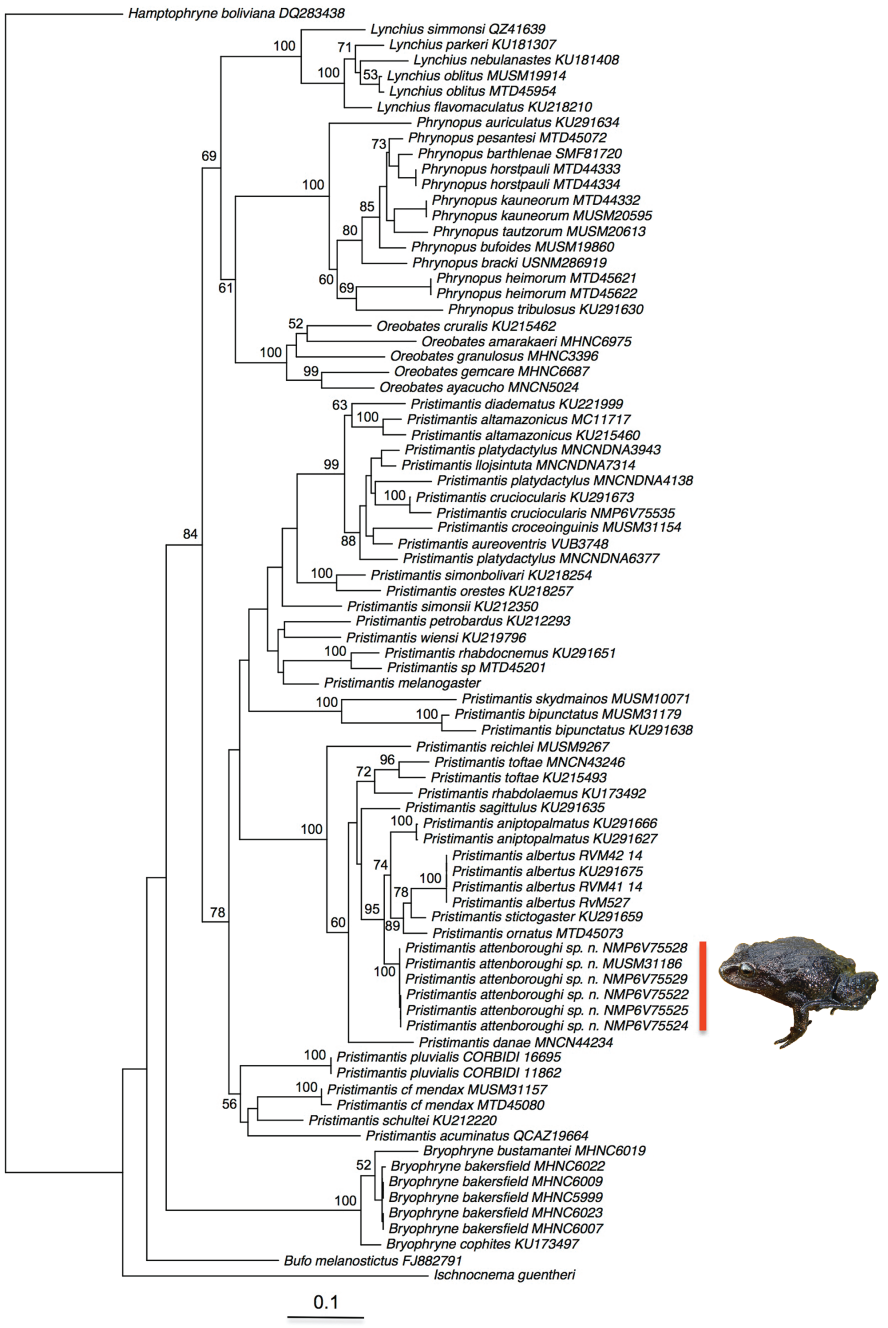
Maximum Likelihood (ML) phylogeny based on the combined 16S + 12S dataset (ML bootstrap values >50 are indicated at each node).

**Molecular phylogenetic analysis.** The Maximum Likelihood (ML) tree (Fig. 2) was generally congruent with a previous molecular phylogeny (Padial et al. 2014) and supported the distinctiveness of the new species from other closely related taxa. Placement of *Pristimantis attenboroughi* sp. n. in the genus *Pristimantis* Jiménez de la Espada, 1871 was strongly supported and, based on the available data, the new species is most closely related to *P. albertus* Duellman and Hedges, 2007, *P. aniptopalmatus* (Duellman and Hedges, 2005), *P. ornatus* (Lehr, Lundberg, Aguilar, and von May, 2006), and *P. stictogaster* (Duellman and Hedges, 2005) (Fig. 2). Table 2 compares uncorrected p-distances of a 542 bp (including gaps) fragment of the 16S mitochondrial rRNA gene of *Pristimantis* species included in our analyses. The lowest distance occurs between the new species and *P. aniptopalmatus* (uncorrected p-distance 4.3 %) while the uncorrected p-distances between the new species and the other three species in the same clade of the *Pristimantis danae* species Group (*P. albertus*, *P. ornatus*, *P. reichlei* Padial and De la Riva, 2009, *P. rhabdolaemus* [Duellman, 1978a], *P. stictogaster* [Duellman and Hedges, 2005], *P. sagittulus* [Lehr, Aguilar, and Duellman, 2004], *P. toftae* [Duellman, 1978b]) vary between 5.2 to 11.8 %.

**Table 2.** Uncorrected p-distances of the 16s mitochondrial rRNA gene for six specimens of *Pristimantis attenboroughi* sp. n. (in bold) and other *Pristimantis* species from GenBank.

		1	2	3	4	5	6	7	8	9
1	*Pristimantis albertus* KU291675									
2	*Pristimantis albertus* RvM41_14	0.000								
3	*Pristimantis albertus* RvM42_14	0.000	0.000							
4	*Pristimantis albertus* RvM527	0.000	0.000	0.000						
5	*Pristimantis attenboroughi* sp. n. NMP6V 75522	**0.062**	**0.065**	**0.062**	**0.066**					
6	*Pristimantis attenboroughi* sp. n. NMP6V 75529	**0.062**	**0.065**	**0.062**	**0.066**	0.000				
7	*Pristimantis attenboroughi* sp. n. NMP6V 75524	**0.062**	**0.065**	**0.062**	**0.066**	0.000	0.000			
8	*Pristimantis attenboroughi* sp. n. NMP6V 75525	**0.062**	**0.065**	**0.062**	**0.066**	0.000	0.000	0.000		
9	*Pristimantis attenboroughi* sp. n. MUSM 31186	**0.062**	**0.065**	**0.062**	**0.066**	0.000	0.000	0.000	0.000	
10	*Pristimantis attenboroughi* sp. n. NMP6V 75528	**0.062**	**0.065**	**0.062**	**0.066**	0.000	0.000	0.000	0.000	0.000
11	*Pristimantis ornatus* MTD45073	0.056	0.059	0.056	0.059	**0.052**	**0.052**	**0.052**	**0.052**	**0.052**
12	*Pristimantis stictogaster* KU291659	0.041	0.043	0.041	0.043	**0.049**	**0.049**	**0.049**	**0.049**	**0.049**
13	*Pristimantis aniptopalmatus* KU291627	0.056	0.059	0.056	0.059	**0.043**	**0.043**	**0.043**	**0.043**	**0.043**
14	*Pristimantis aniptopalmatus* KU291666	0.056	0.059	0.056	0.059	**0.043**	**0.043**	**0.043**	**0.043**	**0.043**
15	*Pristimantis rhabdolaemus* KU173492	0.093	0.097	0.093	0.097	**0.058**	**0.058**	**0.058**	**0.058**	**0.058**
16	*Pristimantis toftae* KU215493	0.110	0.115	0.110	0.115	**0.074**	**0.074**	**0.074**	**0.074**	**0.074**
17	*Pristimantis toftae* MNCN43246	0.105	0.110	0.105	0.110	**0.070**	**0.070**	**0.070**	**0.070**	**0.070**
18	*Pristimantis sagittulus* KU291635	0.093	0.097	0.093	0.099	**0.066**	**0.066**	**0.066**	**0.066**	**0.066**
19	*Pristimantis danae* MNCN44234	0.116	0.121	0.116	0.122	**0.094**	**0.094**	**0.094**	**0.094**	**0.094**
20	*Pristimantis reichlei* MHNSM9267	0.132	0.135	0.132	0.136	**0.118**	**0.118**	**0.118**	**0.118**	**0.118**
		10	11	12	13	14	15	16	17	18	19
1	*Pristimantis albertus* KU291675										
2	*Pristimantis albertus* RvM41_14										
3	*Pristimantis albertus* RvM42_14										
4	*Pristimantis albertus* RvM527										
5	*Pristimantis attenboroughi* sp. n. NMP6V 75522										
6	*Pristimantis attenboroughi* sp. n. NMP6V 75529										
7	*Pristimantis attenboroughi* sp. n. NMP6V 75524										
8	*Pristimantis attenboroughi* sp. n. NMP6V 75525										
9	*Pristimantis attenboroughi* sp. n. MUSM 31186										
10	*Pristimantis attenboroughi* sp. n. NMP6V 75528										
11	*Pristimantis ornatus* MTD45073	**0.052**									
12	*Pristimantis stictogaster* KU291659	**0.049**	0.037								
13	*Pristimantis aniptopalmatus* KU291627	**0.043**	0.048	0.049							
14	*Pristimantis aniptopalmatus* KU291666	**0.043**	0.048	0.049	0.000						
15	*Pristimantis rhabdolaemus* KU173492	**0.058**	0.082	0.076	0.074	0.074					
16	*Pristimantis toftae* KU215493	**0.074**	0.091	0.091	0.083	0.083	0.070				
17	*Pristimantis toftae* MNCN43246	**0.070**	0.099	0.088	0.082	0.082	0.074	0.055			
18	*Pristimantis sagittulus* KU291635	**0.066**	0.084	0.080	0.068	0.068	0.066	0.078	0.095		
19	*Pristimantis danae* MNCN44234	**0.094**	0.107	0.107	0.100	0.100	0.082	0.101	0.100	0.083	
20	*Pristimantis reichlei* MHNSM9267	**0.118**	0.124	0.113	0.117	0.117	0.103	0.126	0.114	0.117	0.113

### Pristimantis attenboroughi sp. n.

Taxon classificationAnimaliaAnuraCraugastoridae

http://zoobank.org/DCE88D49-0EB1-4DA4-A672-5341763B3236

#### Common name.

English: Attenborough’s Rubber Frog. Spanish: Rana cutín Attenborough.

**Figure 3.**
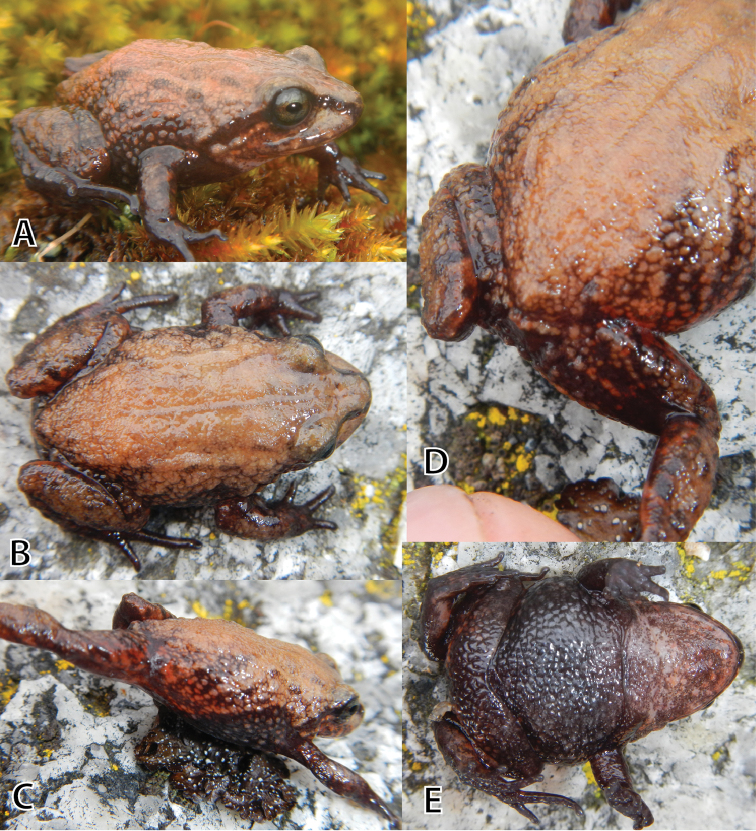
Life male holotype (MUSM 31196, SVL 18.9 mm) of *Pristimantis attenboroughi* sp. n. in dorsolateral view (**A**), dorsal view (**B**), flanks, groin, anterior surfaces of thighs (**C**), posterior surfaces of thighs (**D**), and ventral view (**E**). Photos by E. Lehr.

**Figure 4.**
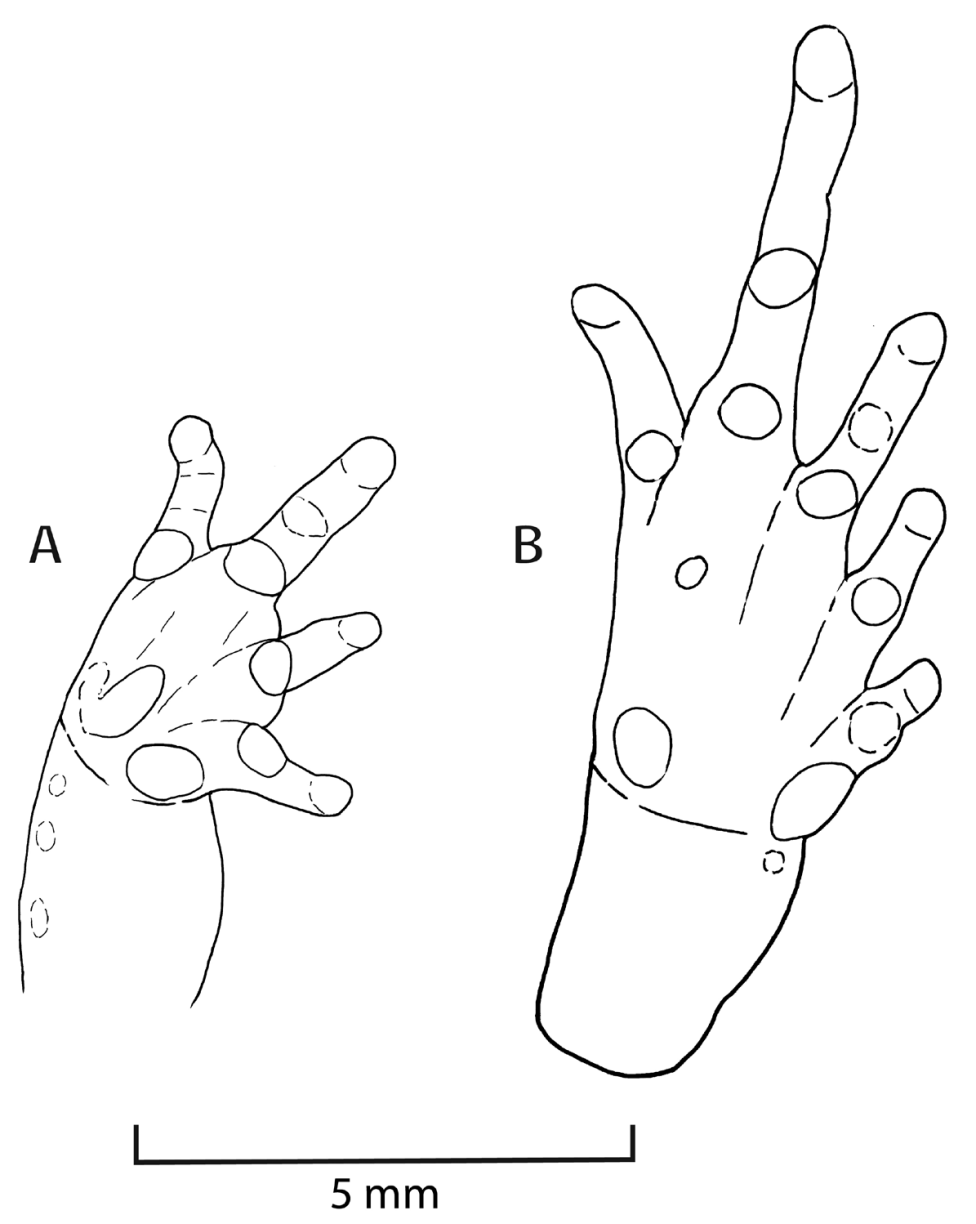
Ventral views of right hand (**A**) and right foot (**B**) of holotype of *Pristimantis attenboroughi* sp. n. (MUSM 31196). Drawings by E. Lehr.

**Holotype.** MUSM 31196 (IWU 178, Figs 3, 4), adult male from the Pui Pui Protected Forest, Provincia Satipo, Región Junín, Peru, Upper part of Quebrada Tarhuish, “Laguna Udrecocha”, Puna, open area on east side of Laguna Udrecocha, 11°23'24.1"S, 74°58'32.5"W, 3936 m a.s.l. (Fig. 8A), collected on 17 May 2012 by E. Lehr and R. von May.

**Figure 5.**
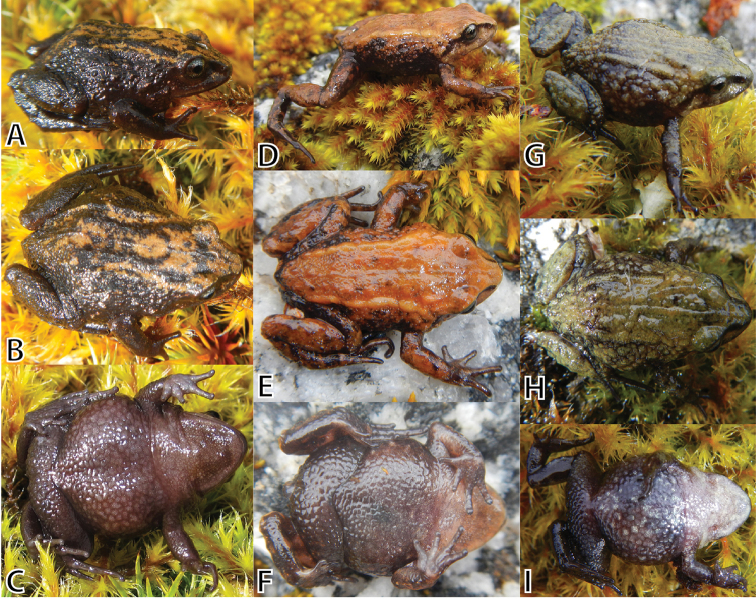
Variation of male paratypes of *Pristimantis attenboroughi* sp. n. in dorsolateral, dorsal, and ventral views. **A–C** (MUSM 31186, SVL 18.6 mm) **D–F** (MUSM 31195, SVL 16.9 mm) **G–I** (MUSM 31992, SVL 15.9 mm). Photos by E. Lehr.

**Figure 6.**
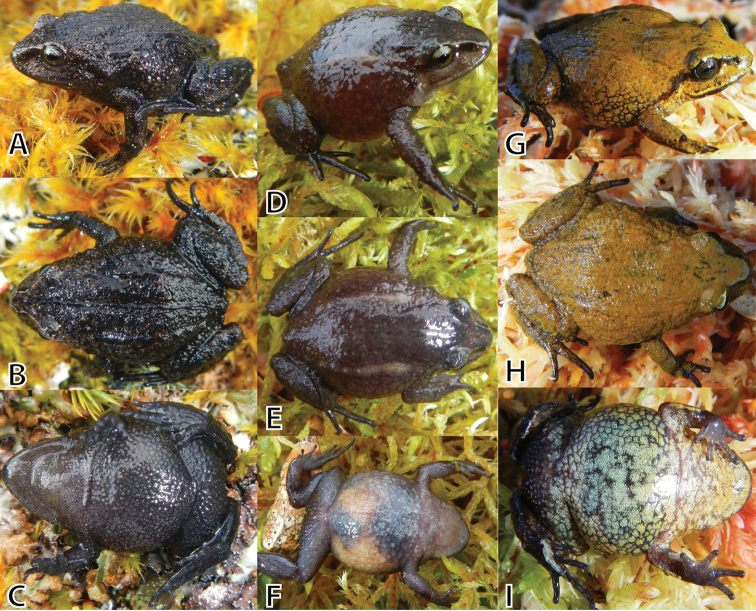
Variation of female paratypes of *Pristimantis attenboroughi* sp. n. in dorsolateral, dorsal, and ventral views. **A–C** (NMP6V 75522, SVL 19.2 mm) **D–F** (MUSM 31987, SVL 23.0 mm) **G–I** (MUSM 31977, SVL 21.9 mm). Photos by E. Lehr.

**Figure 7.**
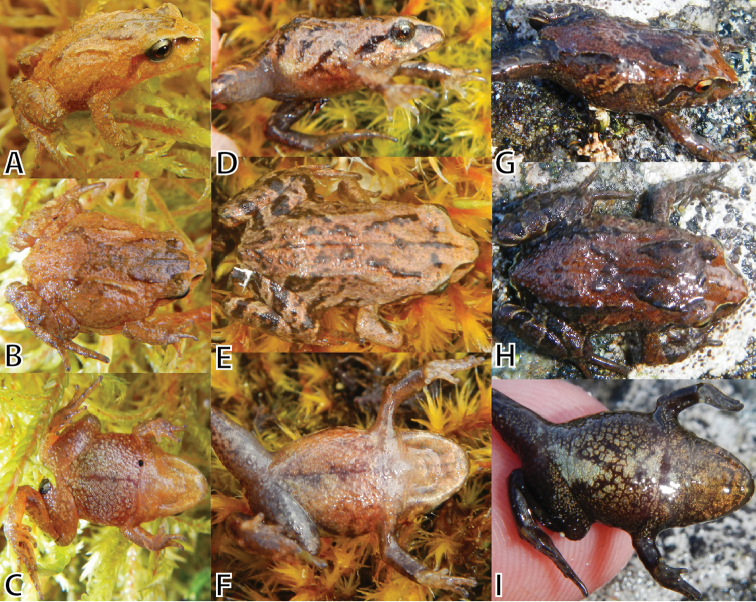
Variation of juvenile paratypes of *Pristimantis attenboroughi* sp. n. in dorsolateral, dorsal, and ventral views. **A–C** (MUSM 31990, SVL 14.0 mm) **D–F** (MUSM 31187, SVL 12.5 mm) **G–I** (MUSM 31200, SVL 14.0 mm). Photos by E. Lehr.

**Figure 8.**
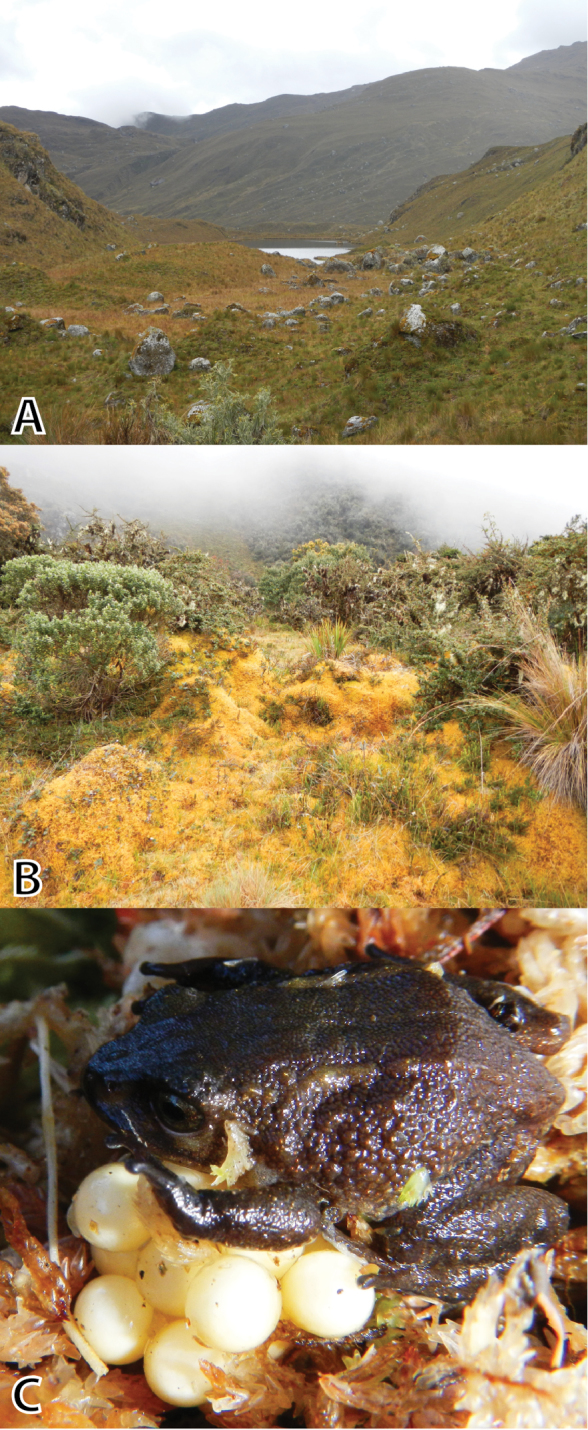
Habitats of *Pristimantis attenboroughi* sp. n. in the PPPF: **A** type locality in the upper Tarhuish valley at Laguna Udrecocha, Puna at 3936 m a.s.l., 17 May 2012 **B** upper montane forest at 3550 m a.s.l. where *P. attenboroughi* sp. n. was found in moss pads **C** female *P. attenboroughi* sp. n. (MUSM 31980, SVL 21.5 mm) guarding a clutch in a moss pad. Photos by E. Lehr.

**Paratypes.** A total of 33 (Figs 5–7, 8C), all from inside the PPPF (except for: MUSM 31199–31202, NMP6V 75526–29), Provincia Satipo, Región Junín: 10 adult females (MUSM 31977, 31980, 31987, 31201, NMP6V 75076, 75522 [GenBank accession numbers KY594753, KY594761], 75523, 75528 [GenBank accession numbers KY594756, KY594764], 75529 [GenBank accession number KY594757], 75534), 20 adult males (MUSM 31186 [GenBank accession number KY594752], 31195, 31199, 31202, 31975, 31979, 31988, 31989, 31992, 31993, NMP6V 75077–75079, 75524 [GenBank accession numbers KY594754, KY594762], 75525 [GenBank accession numbers KY594755, KY594763], 75526, 75527, 75533, UMMZ 244726, 244727), 3 juveniles (MUSM 31187, 31990, 31200).

MUSM 31186, MUSM 31187, NMP6V 75522, 75523: Quebrada Tarhuish, left bank of Antuyo River, “Shiusha”, upper montane forest, 11°22'3.9"S, 74°56'12.7"W, 3414 m a.s.l. collected on 12 May 2012 by E. Lehr and R. von May. MUSM 31195, NMP6V 75524, 75524: collected at the type locality along with the holotype. MUSM 31199, 31200, MUSM 31201, 31202, NMP6V 75526, 75527: Upper part of Quebrada Tasta, “Laguna Luichococha”, Puna, 11°27'23.7"S, 74°55'10.6"W, 3708 m a.s.l. collected on 20 May 2012 by E. Lehr and R. von May. NMP6V 75528, 75529: near trail from Tasta to Tarhuish (first mountain peak), Polylepis forest patch, 11°26'8.6"S, 74°53'56.5"W, 3886 m a.s.l. collected on 20 May 2012 by E. Lehr and R. von May. MUSM 31975: Antuyo, 11°20'03.7"S, 74°59'49.1"W, 3700 m a.s.l. collected on 27 June 2013 by E. Lehr, J. Moravec, and J.C. Cusi. MUSM 31977, 31979, MUSM 31980, NMP6V 75076, UMMZ 244726: Hatunpata, 11°18'07.9"S, 75°01'35.0"W, 3710 m a.s.l. collected on 28 June 2013 by by E. Lehr, J. Moravec, and J.C. Cusi. MUSM 31987–31990, NMP6V 75077, 75078, 75533, UMMZ 244727: Trancapampa, 11°17'49.2"S, 75°00'46.3"W, 3550 m a.s.l. collected on 2 July 2013 by E. Lehr, J. Moravec, and J.C. Cusi. MUSM 31992, 31993, NMP6V 75079, 75534: Antuyo Bajo, 11°18'53.4"S, 74°59'34.8"W, 3400 m a.s.l. collected on 4 July 2013 by E. Lehr, J. Moravec, and J.C. Cusi.

#### Generic placement.

We assign this species to *Pristimantis* based on our molecular data (Fig. 2).

**Diagnosis.** A new species of *Pristimantis* assigned to the *danae* species Group having the following combination of characters: (1) Skin on dorsum shagreen with low scattered tubercles, skin on flanks tuberculate, skin on venter areolate; discoidal fold absent, thoracic fold present; irregularly shaped, discontinuous dorsolateral folds present; (2) tympanic membrane and tympanic annulus absent; (3) snout short, rounded in dorsal and in lateral views; (4) upper eyelid without enlarged conical tubercles; EW shorter than IOD; cranial crests absent; (5) dentigerous processes of vomers present; (6) males without vocal slits, nuptial pads absent; (7) Finger I shorter than Finger II; tips of digits narrow, rounded, lacking circumferential grooves; (8) fingers without lateral fringes; (9) small conical ulnar and tarsal tubercles present; (10) heel with a small conical tubercle; inner tarsal fold usually absent; (11) inner metatarsal tubercle ovoid, 1.5 times as large as outer; outer metatarsal tubercle small, rounded; vie low supernumerary plantar tubercles; (12) toes without lateral fringes; basal toe webbing absent; Toe V longer than Toe III; tips of digits narrow, rounded, lacking circumferential grooves, toe tips slightly smaller than those on fingers; (13) in life, dorsal ground coloration pale or dark gray, reddish brown or brownish olive with dark gray scattered flecks, some with X-shaped mark on scapular and ill-defined diagonal bars on flanks; dark grayish-brown canthal and supratympanic stripes usually present; groin dark gray or pale reddish brown with a pale red to pink tint in some; venter dark gray, pale gray, grayish brown or pale grayish green and in some dark gray mottled; iris pale grayish green with fine black vermiculation and brownish-orange horizontal streak across pupil and lower half of iris; (14) SVL in adult males 14.6–19.2 mm (n = 21), in adult females 19.2–23.0 mm (n = 10).

**Comparisons.** *Pristimantis attenboroughi* is readily distinguished from its congeners in Ecuador (176 species, AmphibiaWeb 2016), Peru (128 species, AmphibiaWeb 2016), and Bolivia (17 species, AmphibiaWeb 2016) by having narrow digits without circumferential grooves, by lacking a tympanic annulus and tympanic membrane, and by having irregularly shaped, discontinuous dorsolateral folds. In Peru 18 species of *Pristimantis* lack a tympanum; these are *P. academicus* Lehr, Moravec, and Gagliardi Urrutia, 2010, *P. altamazonicus* (Barbour and Dunn, 1921), *P. ashaninka* Lehr and Moravec, 2017, *P. colodactylus* (Lynch, 1979), *P. coronatus* Lehr and Duellman, 2007a, *P. croceoinguinis* (Lynch, 1968), *P. cruciocularis* (Lehr, Lundberg, Aguilar, and von May, 2006), *P. flavobracatus* (Lehr, Lundberg, Aguilar, and von May, 2006), *P. imitatrix* (Duellman, 1978b), *P. lirellus* (Dwyer, 1995), *P. leucorrhinus* Boano, Mazzotti, and Sindaco, 2008, *P. martiae* (Lynch, 1974), *P. minutulus* Duellman and Hedges, 2007, *P. rhabdocnemus* (Duellman and Hedges, 2005), *P. simonsii* (Boulenger, 1900), *P. tantanti* (Lehr, Torres-Gastello, and Suárez-Segovia, 2007), *P. ventrimarmoratus* (Boulenger, 1912), and *P. vilcabambae* Lehr, 2007. Of these, only *Pristimantis simonsii* from northern Peru has narrow digits without circumferential grooves. *Pristimantis attenboroughi* and *P. simonsii* lack circumferential grooves and a tympanum, and both have dorsolateral folds, but *P. attenboroughi* is smaller than *P. simonsii* (female SVL 26.2–33.3 mm in *P. simonsii*), and male *P. attenboroughi* lack nuptial pads which are present in *P. simonsii*.

Members of the *Pristimantis orestes* species Group are terrestrial and inhabit high elevations in southern Ecuador and in Peru (Duellman and Lehr, 2009) and have narrow digits, and only one of the 17 species (Guayasamin and Artega 2013) lacks circumferential grooves (*P. simonsii*), and only two (*P. seorsus, P. simonsii*) lack a tympanum. Furthermore *P. attenboroughi* is phylogenetically distant from members of this group which is considered to be not monophyletic (Duellman and Lehr 2009, Fig. 2).

Among the three other new species of *Pristimantis* from the upper montane forests and Puna of the PPPF, only *Pristimantis* sp. n. E lacks circumferential grooves and a tympanum. However, *P. attenboroughi* and *P.* sp. n. E both differ regarding other morphological traits, coloration, and genetically.

*Pristimantis attenboroughi* shares with *P. stipa* Venegas and Duellman, 2012 from the Puna of northern Peru (Venegas and Duellman 2012) narrow digits without circumferential grooves and dorsolateral folds. However, *P. attenboroughi* is smaller (female SVL 19.2–23.0 mm [n = 10] vs. 35.1 mm [n = 1]), lacks a tympanum (present in *P. stipa*), and has ulnar tubercles not coalesced into fold (coalesced into low fold in *P. stipa*), Venegas and Duellman (2012).

The new species shares narrow digits without circumferential grooves and the absence of a tympanic annulus and tympanic membrane with the Andean genera *Phrynopus* Peters, 1873 (except for *Phrynopus auriculatus* Duellman and Hedges, 2008, and *P. peruanus* Peters, 1873), 28 species from elevations between 2200 and 4400 m a.s.l. in central and northern Peru, Duellman and Lehr, 2009) and *Bryophryne* Hedges, Duellman, and Heinicke, 2008 (8 species from elevations between 2900 and 4120 m a.s.l. in southern Peru, Duellman and Lehr 2009), AmphibiaWeb (2016). *Pristimantis attenboroughi* is most similar with *Phrynopus chaparroi* Mamani and Malqui, 2014 which was described based on morphological characters and found at elevations between 4205 and 4490 m a.s.l. in southern Región Junín (Mamani and Malqui 2014). Both *Pristimantis attenboroughi* and *Phrynopus chaparroi* lack a tympanum and have narrow digits without circumferential grooves. However, *P. attenboroughi* is smaller than *P. chaparroi* (female SVL 19.2–23.0 mm [n = 10] vs. 30.0–32.2 [n = 4]), lacks protuberant subconical postrictal tubercles (present in *P. chaparroi*), has dorsolateral folds (absent in *P. chaparroi*), dentigerous processes of vomers present (absent in *P. chaparroi*), and males lack nuptial pads (present in *P. chaparroi*). *Phrynopus chaparroi* might belong to *Pristimantis*, but molecular characters need to be applied to confirm our suspicion.

#### Description of the holotype.

Head about as long as wide; head length 39.7% of SVL; head width 38.6% of SVL; cranial crests absent; snout short, rounded in dorsal view, rounded in lateral view (Fig. 3A, B); eye-nostril distance 70% of eye diameter; nostrils slightly protuberant, directed dorsolaterally; canthus rostralis short, rounded in lateral view, weakly concave in dorsal view; loreal region concave; lips rounded; outer margin of upper eyelid each with few slightly enlarged conical tubercles; upper eyelid width 51.9% of IOD (see photo in life Fig. 3); supratympanic fold short and broad, extending from posterior margin of upper eyelid slightly curved to insertion of arm; tympanic membrane and annulus absent; distinct conical postrictal tubercles present bilaterally. Choanae small, ovoid, not concealed by palatal shelf of maxilla; dentigerous processes of vomers positioned posterior to level of choanae, oblique, narrowly separated; tongue long, oval, about three times as long as wide, not notched posteriorly, posterior half free.

Skin on dorsum shagreen with low scattered tubercles, skin on flanks tuberculate, irregularly shaped, discontinuous dorsolateral folds present extending from posterior level of tympanic area to level of hind limb insertion; skin on throat, chest, and belly areolate; discoidal fold absent, thoracic fold present; cloacal sheath short.

Outer ulnar surface each with a row of four minute low tubercles; palmar tubercle bifid; thenar tubercle ovoid; subarticular tubercles well defined, most prominent on base of fingers, round in ventral view, subconical in lateral view; supernumerary tubercles indistinct; fingers short and stout lacking lateral fringes, Finger I shorter than Finger II; tips of digits of fingers narrow, round, lacking circumferential grooves (Fig. 4A).

Hind limbs short, slender, tibia length 40.2% of SVL; foot length 41.3% of SVL; dorsal surfaces of hind limbs tuberculate; inner surface of thighs smooth, posterior surfaces of thighs tuberculate, ventral surfaces of thighs areolate; heels each with a small conical tubercle; outer surface of tarsus with few scattered minute low tubercles; inner tarsal fold absent, but small tubercle proximal to metatarsal tubercle; inner metatarsal tubercle ovoid, one and a half times the size of round outer metatarsal tubercle; subarticular tubercles well defined, round in ventral view, subconical in lateral view; few plantar supernumerary tubercles, about one third the size of subarticular tubercles; toes without lateral fringes; basal webbing absent; tips of digits narrow, round, less expanded than those on fingers, lacking circumferential grooves; relative length of toes: 1<2<5<3<4; Toe V slightly longer than Toe III (tip of digit of Toe III and Toe V not reaching distal subarticular tubercle on Toe IV; Fig. 4B).

#### Measurements (in mm) of the holotype.

SVL 18.9; tibia length 7.6; foot length 7.8; head length 7.5; head width 7.3; eye diameter 2.0; inter orbital distance 2.7; upper eyelid width 1.4; internarial distance 1.9; eye–nostril distance 1.4.

#### Coloration of the holotype in life

**(Fig. 3).** The dorsal ground coloration is pale reddish brown with few dark brown flecks; narrow dark brown canthal and supratympanic stripes; flanks pale reddish brown with dark brown flecks forming irregularly shaped diagonal bars; groin and anterior surfaces of thighs reddish brown with dark brown flecks and pale reddish tint; chest, belly, and ventral surfaces of thighs dark grayish brown, throat pale reddish brown and pale gray mottled; palmar and plantar surfaces, and fingers and toes dark grayish brown; iris pale grayish green with fine black vermiculation and brownish-orange horizontal streak across pupil and lower half of iris.

**Table 3.** Measurements (in mm) of selected adult type specimens of *Pristimantis attenboroughi* sp. n. M = male, F = female. For other abbreviations see methods.

Characters	MUSM 31988	MUSM 31992	MUSM 31186	UMMZ 244727	NMP6V 75523	MUSM 31980	MUSM 31977	NMP6V 75076	MUSM 31987
sex	M	M	M	M	F	F	F	F	F
SVL	14.6	15.9	18.6	19.2	20.1	21.5	21.9	22.9	23.0
TL	6.0	6.2	7.3	6.8	8.3	8.4	8.1	8.3	8.8
FL	5.8	6.1	7.7	7.3	9.4	8.8	8.8	9.2	10.2
HL	5.3	6.2	6.2	6.8	7.5	7.6	7.3	8.4	7.1
HW	5.0	5.7	6.3	6.6	7.4	7.8	7.8	7.9	7.9
ED	1.6	1.7	1.9	1.9	2.0	2.2	2.4	2.4	2.2
IOD	1.8	2.1	2.4	2.1	2.7	2.5	2.3	2.6	2.9
EW	0.9	1.4	1.2	1.3	1.6	1.6	1.6	1.6	1.3
IND	1.3	1.5	1.7	2.0	2.0	1.9	2.1	2.3	2.1
E-N	1.1	1.0	1.3	1.3	1.3	1.7	1.5	1.8	1.7

#### Coloration of the holotype in preservative.

The dorsal ground coloration is pale brown with few dark brown flecks; narrow dark brown canthal and supratympanic stripes; flanks pale brown with many dark brown flecks forming irregularly shaped diagonal bars; groin and anterior surfaces of thighs brown with dark brown flecks; chest, belly, and ventral surfaces of thighs dark brown, throat pale brown and pale gray mottled; palmar and plantar surfaces, and fingers and toes dark brown; iris pale gray.

**Table 4.** Measurements (in mm) and proportions of adult male and adult female type specimens of *Pristimantis attenboroughi* sp. n.; ranges followed by means and one standard deviation in parentheses. For abbreviations see methods.

**Characters**	**Males (n = 21)**	**Females (n = 10)**
SVL	14.6–19.2 (17.1 ± 1.2)	19.2–23.0 (21.6 ± 1.1)
TL	5.8–7.6 (6.7 ± 0.5)	8.0–8.8 (8.4 ± 0.2)
FL	5.8–7.8 (7.0 ± 0.5)	8.8–10.2 (9.3 ± 0.4)
HL	5.3–7.3 (6.3 ± 0.5)	7.1–8.4 (7.6 ± 0.4)
HW	5.0–6.9 (6.0 ± 0.5)	7.3–8.3 (7.9 ± 0.3)
ED	1.6–2.1 (1.9 ± 0.2)	1.8–2.4 (2.1 ± 0.2)
IOD	1.8–2.5 (2.1 ± 0.1)	2.3–2.9 (2.7 ± 0.2)
EW	0.9–1.9 (1.3 ± 0.2)	1.3–1.7 (1.5 ± 0.1)
IND	1.3–2.1 (1.6 ± 0.2)	1.9–2.3 (2.1 ± 0.1)
E-N	0.8–1.4 (1.2 ± 0.1)	1.3–1.8 (1.5 ± 0.2)
TL/SVL	0.34–0.44	0.36–0.42
FL/SVL	0.35–0.46	0.40–0.47
HL/SVL	0.33–0.41	0.31–0.39
HW/SVL	0.31–0.38	0.34–0.39
HW/HL	0.84–1.02	0.94–1.11
E-N/ED	0.47–0.71	0.62–0.89
EW/IOD	0.45–0.70	0.45–0.70

**Variation.** All paratypes (Figs 5–7) are similar to the holotype regarding morphology and proportions (Tables 3, 4). Besides differences in SVL, notable morphological variation includes prominence of dorsolateral folds (e.g., prominent dorsolateral folds in MUSM 31192, 31195, Fig. 5D–F, G–I; weak dorsolateral folds in MUSM 31186, 31975, 31977, NMP6V 75522, 75528, 75529, Fig. 6G–I), and coarseness of tuberculate skin texture on flanks and hind limbs (skin coarsely tuberculate in MUSM 31186, 31192, 31195, NMP6V 75525, Fig. 5; skin weakly tubercular MUSM 31987, 31997, NMP6V 75528, 75529). Two specimens (NMP6V 75529, 75534) have a tubercle-like inner tarsal fold present. *Pristimantis attenboroughi* demonstrates a remarkable polymorphism in coloration (Figs 5–7).

The dorsal coloration ranges from pale gray (MUSM 31987, NMP6V 75533, Fig. 6D–F), dark gray (MSUM 31186, 3199, NMP6V 75522, 75523, 75528, 75529, Fig. 6A–C), reddish brown (MUSM 31195, 31975, NMP6V 75525, Figs 5D–F) to brownish olive (MUSM 31992, 31997, Figs 5G–I, 6G–I) with dark gray scattered flecks. Some have an X-shaped mark on scapular (MUSM 31200, 31975, 31990), some ill-defined diagonal bars on the flanks (MUSM 31195). Dark grayish-brown canthal and supratympanic stripes are usually present except for dark gray specimens (MSUM 31186, 3199, NMP6V 75522, 75523, 75528, 75529). The groin is dark gray (MSUM 31186, 3199, NMP6V 75522, 75523, 75528, 75529) or pale reddish brown with a pale red to pink tint in some specimens (MUSM 31195, 31196). The venter is dark gray (NMP6V 75522, 75523, 75528, 75529, Fig. 6C), pale gray (MUSM 31987, Fig. 6F), grayish brown (MUSM 31186, 31195, NMP6V 75525, Fig. 5C, F) or pale grayish green and gray mottled (MUSM 31197, Fig. 6I) or dark gray and pale gray mottled (MUSM 31199, 31975, 31992, NMP6V 75533, Fig. 5I).

Juveniles (MUSM 31187, 31990, 31200, Fig. 7) have a paler coloration (yellowish to reddish brown) with contrasting dark brown flecks and distinct canthal and supratympanic stripes. All have the iris pale grayish green with fine black vermiculation and brownish-orange horizontal streak across pupil and lower half of iris, and usually a narrow vertical dark gray streak from pupil through middle of lower iris.

#### Etymology.

We dedicate this species to Sir David Frederick Attenborough in honor for his educational documentaries on wildlife, especially on amphibians (e.g., *Life in Cold Blood, Fabulous Frogs*), and for raising awareness about the importance of wildlife conservation. The specific epithet is used as noun in apposition.

#### Distribution, natural history, and conservation status.

**Figure 9.**
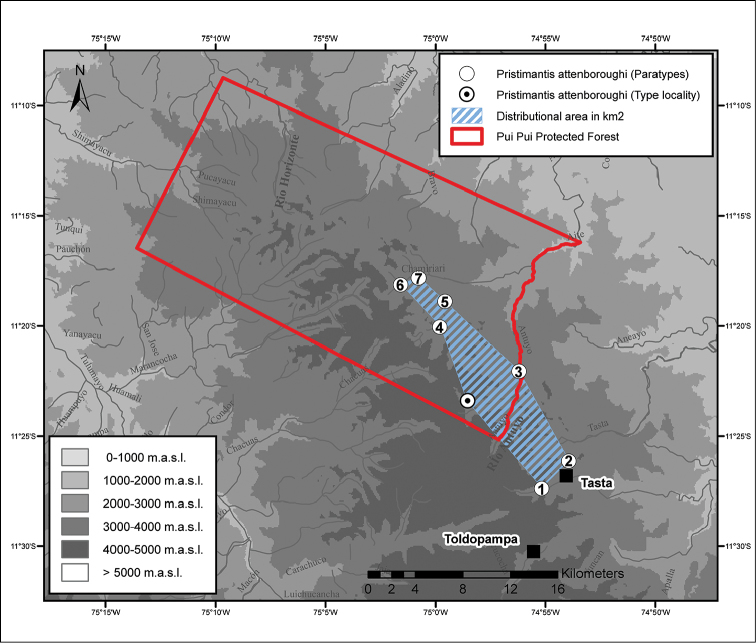
Distribution of *Pristimantis attenboroughi* sp. n. in the PPPF and its surroundings: type locality: Laguna Udrecocha, 3936 m a.s.l.; **1** Upper part of Quebrada Tasta, “Laguna Luichococha”, 3708 m a.s.l. **2** near trail from Tasta to Tarhuish (first mountain peak), *Polylepis* forest patch, 3886 m a.s.l. **3** Quebrada Tarhuish, left bank of Antuyo River, “Shiusha”, 3414 m a.s.l. **4** Antuyo, 3700 m a.s.l. **5** Antuyo Bajo, 3400 m a.s.l. **6** Hatunpata, 3710 m a.s.l. **7** Trancapampa, 3550 m a.s.l.

*Pristimantis attenboroughi* is known from six localities inside the PPPF (Puna of Quebrada Tarhuish at Laguna Udrecocha, Fig. 8A; upper montane forest of Quebrada Tarhuish on the left bank “Shiusha” of Antuyo River; Antuyo; Antuyo Bajo; Hatunpata, and Trancapampa, Figs 8B, 9) and from two outside the PPPF (upper part of Quebrada Tasta close to Laguna Luichococha; in *Polylepis* forest of first mountain peak next to trail from Tasta to Tarhuish), and is distributed at elevations between 3400 and 3936 m a.s.l., Fig. 9. The type locality (Figs 8A, 9), upper part of Quebrada Tarhuish, on the east side of Laguna Udrecocha at 3936 m a.s.l., belongs to the Puna ecoregion (Brack 1986). The vegetation consists of Peruvian feather grass (*Stipa ichu*), mosses, and small bushes. The holotype was found inside moss in the afternoon on 17 May 2012. No sympatric anurans were found at the type locality. At the upper montane forest of Quebrada Tarhuish on the left bank “Shiusha” of Antuyo River, *P. attenboroughi* was found deep inside large moss layers. Sympatric anurans are *Gastrotheca griswoldi* (MUSM 31193), *Pristimantis* sp. n. C (MUSM 31190–92), *Pristimantis* sp. n. D (MUSM 31197–98), and *Phrynopus* sp. n. A (MUSM 31203).

A female *Pristimantis attenboroughi* (MUSM 31980, Fig. 8C) guarding 20 eggs was found at Hatunpata inside moss, 3710 m a.s.l., on 28 June 2013. The eggs were pale cream colored and had an average diameter of 3.5 ± 0.1 mm (3.3–3.6 mm, n = 20).

The IUCN Red List criteria (IUCN 2001) consider that if a species occurs in fewer than 10 threat-defined locations and the extent of occurrence (EOO) is < 20,000 km^2^, it should be classified as Vulnerable or Endangered. *Pristimantis attenboroughi* is known from seven localities distributed in the PPPF and its buffer zone (Fig. 9), with an estimated EOO of 66.54 km^2^. As such, this new species might be classified as Vulnerable if we take into account these criteria. However, given that the PPPF may host a greater number of locations and most of them are inside the protected area, we propose that *Pristimantis attenboroughi* should likely be categorized as Near Threatened (NT).

Given that the known distribution of *Pristimantis attenboroughi* overlaps with the PPPF, a substantial portion of the habitat of this species is formally protected. However, other factors such as fungal infections, climate change, pollution, and man-made fires (used to expand grazing areas for livestock) continue to be threats for many Andean amphibians even inside protected areas (Catenazzi and von May 2014).

## Discussion

When we encountered the first specimen of *Pristimantis attenboroughi* in the field both of us were sure that we had found a new species of *Phrynopus* because of its overall morphological appearance: most species in the genus *Phrynopus* usually lack tympanum, have narrow digits without circumferential grooves and are distributed at high elevations. However, following an integrative taxonomy approach that included molecular and morphological data, we realized that *Pristimantis attenboroughi* is not a *Phrynopus* species. Our analysis also revealed that *Pristimantis attenboroughi* is not closely related to other *Pristimantis* species that have narrow digits (e.g., members of the *P. orestes* species group), an assumption that could have been made if only morphological data were available. In other words, *Pristimantis attenboroughi* displays convergence that easily could have led to an incorrect generic assignment. *Pristimantis attenboroughi* is morphologically most similar to *Phrynopus chaparroi* (Mamani and Malqui 2014) and we assume that the latter species might belong to *Pristimantis* and to the *danae* species group. Thus, molecular data are needed to determine whether the current generic placement of *Phrynopus chaparroi* is correct.

With *Pristimantis attenboroughi*, seven species of *Pristimantis* are known from the Puna (> 3000 m a.s.l.) of Peru. Of these, six occur in northern Peru (*P. atrabracus* [Duellman and Pramuk, 1999], 2963–3330 m a.s.l.; *P. bellator* Lehr, Aguilar, Siu-Ting, Jordán, 2007, 1900–3100 m a.s.l.; *P. cordovae* [Lehr and Duellman, 2007b], 3400–4100 m a.s.l.; *P. mariaelenae* Venegas and Duellman, 2012, 3596 m a.s.l.; *P. pinguis* [Duellman and Pramuk, 1999], 3000–3916 m a.s.l.; *P. stipa* Venegas and Duellman, 2012, 3596 m a.s.l.), and only one species in central Peru (*P. attenboroughi*, 3400–3936 m a.s.l.), Duellman and Lehr 2009. Navarrete et al. (2016) pointed out the disparity in species richness of *Pristimantis* at high elevation between Ecuador (18 species of *Pristimantis*) and Peru (5 species of *Pristimantis*). Whilst the Páramo in Ecuador is more humid than the drier Puna in Peru, it is likely that, besides climatic differences between the two regions, the lower species richness of *Pristimantis* in the Puna of Peru is an artifact of lower survey effort and the presence of other high-elevation clades not present in Ecuador. Thus, we hypothesize that the occurrence of the genus *Phrynopus* at high elevations (28 species from elevations between 2200–4400 m a.s.l., AmphibiaWeb 2016, Duellman and Lehr 2009) in central Peru might restrict the number of niches available for *Pristimantis* at high elevations.

Additional new species of terrestrial-breeding frogs from montane forests and Puna of the PPPF will be described in the near future.

## Supplementary Material

XML Treatment for Pristimantis attenboroughi

## Appendix

**Comparative specimens examined**

*Pristimantis mariaelenae*: Peru: Lambayeque: Cañaris, 3406–3494 m: MUSM 26478.

*Pristimantis simonsii*: Peru: Cajamarca: 23.5 km NE Encanada, 3510 m: MUSM 1163–1179.

*Pristimantis stipa*: Peru: Peru: Lambayeque: Cañaris, 3406–3494 m: MUSM 26481, 26482.

*Phrynopus* sp. n. A: Peru: Junín: Pui Pui Protected Forest: near trail from Tasta to Tarhuish (first mountain peak), Polylepis forest patch, 3886 m: MUSM 31203.

*Pristimantis* sp. n. C: Peru: Junín: Pui Pui Protected Forest: Quebrada Tarhuish on the left bank „Shiusha“ of Antuyo River, 3414 m: MUSM 31190–92.

*Pristimantis* sp. n. D: Peru: Junín: Pui Pui Protected Forest: Quebrada Tasta, Runda, 3463 m: MUSM 31197–98.

*Pristimantis* sp. n. E: Peru: Junín: Peru: Junín: Pui Pui Protected Forest: Laguna Sinchon, 3890 m: MUSM 31981–83.
